# Association patterns of female gorillas

**DOI:** 10.1098/rstb.2021.0429

**Published:** 2023-01-16

**Authors:** Christopher Young, Martha M. Robbins

**Affiliations:** ^1^ Department of Psychology, Nottingham Trent University, Nottingham NG1 4FQ, UK; ^2^ Max Planck Institute for Evolutionary Anthropology, Deutscher Platz 6, 04103 Leipzig, Germany

**Keywords:** association patterns, cross-species, female dispersal, social network analysis, social relationship

## Abstract

Social interactions ultimately impact health and fitness in gregarious mammals. However, research focusing on the strength of affiliative interactions has primarily been conducted on female philopatric species. Gorillas provide an interesting counterpoint to previous research as females emigrate multiple times throughout their lives. We compare female–female association strength, duration and consistency in wild mountain (*Gorilla beringei beringei*) and western gorillas (*Gorilla gorilla gorilla*). Additionally, we examine whether the alpha male influences female association strength and if these associations are an artefact of both females concurrently in spatial proximity of the alpha male. In this between-species comparison, female gorillas had differentiated association patterns that were consistent on average for 2 years. The alpha males did not influence female association strength, with associations being similar in his presence or absence. Finally, we found more variability in association patterns among mountain gorillas with higher average association scores and higher proportion of ‘preferred associates' than western gorillas. The rare dispersal pattern in the *Gorilla* genus may lead to greater flexibility in female association patterns than in species exhibiting female philopatry and strong kinship bonds. This may echo ancestral human society and provide new evidence to help us understand the evolution of modern human society.

This article is part of the theme issue ‘Cooperation among women: evolutionary and cross-cultural perspectives’.

## 1. Introduction

For gregarious mammals including humans, social interactions shape daily life and can impact health, physiology and ultimately survival [1–3]. Therefore, it is important to understand the extent to which individuals form and maintain social relationships in group-living species [4,5]. Social relationships result from repeated agonistic and affiliative interactions between two partners [5] and vary from highly differentiated to relatively uniform across group-living mammals [6].

Mammalian females are often philopatric [7,8] and thus remain in their natal group throughout their lifetime. As a result, female kinship patterns are often found to be a major underlying mechanism in the group social structure [9,10]. In such cases, social relationships tend to be highly differentiated and stronger relationships form among kin (and within matrilines) and can be stable over time [6,11]. The formation and maintenance of strong relationships provide several benefits, including the propensity to cooperate in some species, ultimately translating into enhanced fitness [2,12,13].

Female-biased dispersal is less common in mammals [7,8] and consequently the knowledge of female social relationships and their function in such species is limited. Female-biased dispersal can provide benefits, such as reduced feeding competition, infanticide risk and inbreeding probability [14–17], which can outweigh the benefits of residing with kin. Due to the expected lack of kin in a female's new group, social relationships among dispersing females are predicted to be weak and ephemeral in comparison to those of philopatric females [14,16–18]. However, social relationships may carry benefits by enhancing cooperation, such as tolerance during feeding, protection against male aggression or facilitating coalition formation [6,10,16,17]. To date, only a handful of studies have examined female social relationships in species with female dispersal.

Overall, for species with female dispersal, studies reveal a range of social relationship structures from differentiated and stable to more ephemeral and homogeneous. Female Ugandan red colobus monkeys (*Piliocolobus tephrosceles*) show limited affiliative interactions and inconsistency in their social relationships, as predicted for species with female-biased dispersal [19]. By contrast, in several species, the findings often do not support the expectation of weak social relationships. Strong, differentiated relationships have been found among some dyads of female chimpanzees (*Pan troglodytes* [20–22]), black-and-white colobus monkeys (*Colobus vellerosus* [23]) and feral horses (*Equus ferus* [24]). Bonobo (*Pan paniscus*) females have been found to form strong inter- and intra-sexual social relationships [25–27], which differed in duration and stability, while kinship did not predict the relationships between females ([28], also see [29]; wild horses). Despite female kin having stronger relationships than non-kin in chimpanzees, relationship strength among kin did not predict their tendency to cooperation via coalitions whereas those females who formed tolerant relationships (mostly non-kin) did leverage relationships for cooperation [22]. In these species, females typically migrate only once in their lifetime, so they may live in a group with the same social partners for many years and thus investing in long-term social relationships may still be beneficial.

Early hominids are thought to have exhibited some degree of female dispersal [29–33] and thus females may not have resided with female kin. Gorillas may resemble aspects of early hominid grouping patterns due to their complex structure and fluidity of group membership [30,32–36], but long-term evaluations of female dyadic relationships remain understudied (but see [37]). Here we examine female associations as a first step of comparing the social relationships of two closely related apes. We compare and contrast female association consistency, strength and differentiation in three mountain gorilla groups (*Gorilla beringei beringei*) and one western gorilla group (*Gorilla gorilla gorilla*), and the potential influence of the alpha males on female associations.

The two species of gorillas, occupying a wide range of ecological conditions across central Africa, exhibit large differences in dietary patterns but have many similarities in their basic social organization [38]. Females of both species disperse from their natal group and may transfer between social groups multiple times in their lives, such that residing with close kin (mothers, daughters or siblings) is not common ([15,39,40] but see [37]). Emigration rates are higher for western gorilla females than for mountain gorillas, which may be due to female mountain gorillas having more within-group mate choice because they often reside in multi-male groups or due to female western gorillas responding to feeding competition by emigrating to smaller groups [15]. Females are believed to associate with a silverback (adult male) for protection against infanticide from other males and predation [15,39,41,42]. Additionally, male–female social relationships are considered to be the most important among adults, with female–female relationships regarded as weak, but nearly all studies on social relationships have been conducted on one population of mountain gorillas (e.g. [36,40,43,44]). Therefore, we will also examine the role of the alpha male in female–female association patterns.

Here we quantify the strength and consistency of association patterns among females of the two species of gorillas and test predictions for intra- and interspecific differences. We only examine association patterns here because grooming behaviour between female mountain gorillas is irregular, and it is extremely rare among adult western gorillas [45]. Association partner preferences have been found in a range of mammalian species and promote cooperation and shared benefits [27,46–48]. Our first aim is to empirically test if female association strength is an artefact of both females trying to stay in spatial proximity of the alpha male. We examine this because the alpha male is believed to be responsible for group cohesion and male–female social relationships are the strongest among adults [40]. If females associate with each other due to concurrently staying in proximity to the alpha male, we would predict dissimilar association scores in his presence or absence, whereas if he is not driving these associations, we expect similar scores when he is present or absent. Secondly, due to female western gorillas having higher dispersal rates (and hence on average shorter co-residency times with other females), we predict mountain gorillas to have a larger variation in association scores, strong associations to be more common and greater consistency in dyadic association patterns than western gorillas. Understanding these association patterns in two closely related female-biased dispersing species will further advance our understanding of the evolution of both mammalian and complex human sociality.

## 2. Methods

### (a) Study site and animals

Observations were conducted on three groups of habituated mountain gorillas in Bwindi Impenetrable National Park, Uganda. The Kyagurilo (KYA) group was observed from 2001 to 2019 and contained an annual mean of 6.2 females (range = 5–7), Bitukura (BIT) group was observed from 2015 to 2019 with an annual mean of 4 females (range = 3–5) and Oruzogo (ORU) group was observed from 2015 to 2019 and contained an annual mean of 6.6 females (range = 6–8). One group of habituated western gorillas in Loango National Park, Gabon (Atananga Group; ATA) was observed from 2015 to 2019 and contained an annual mean of 5.6 females (range = 3–7)*.* Both ORU and BIT were multi-male throughout the study period, KYA was both one-male and multi-male, while ATA was always one-male. All research assistants collecting data were trained and supervised on a routine basis by MMR to ensure uniformity in data collection. Females were considered as adult when aged 10 years or older and no known mother–adult daughter, full or half-sibling pairs were co-residing during the study period.

### (b) Behavioural data collection

In Bwindi, observations of the gorillas were limited to 4 hours per day, as per regulations of the Uganda Wildlife Authority, and typically occurred between 08.00 and 15.00 h. In Loango, observations were conducted all day, typically between 07.00 and 17.00 h for an average of 5.7 h per day. Data collection protocols were identical at the two study sites and consisted of focal animal sampling and instantaneous scan sampling [49]. Female focal sampling was balanced between individuals across each month. Focal periods were of 15- to 60-min duration, with variation usually arising due to difficulty in following the gorillas in dense understory vegetation and swamps. Concurrent with the focal animal sampling, instantaneous scan sampling was conducted at 10 min intervals, during which the activity of the focal animal was recorded (feeding, resting, travelling or other) as well as the identity, activity and proximity of all other group members in view to the focal animal. We used a distance of less than 5 m to the focal animal as a measure of female association (see [43,44]).

### (c) Social proximity measure

To examine the structure of social associations, we examined spatial proximity. We used the data of when an individual was within 5 m of the focal animal in the instantaneous scan sampling to construct social matrices and examine the dynamics of the social relationship using the ‘netTS’ package [50] in R [51]. This package uses the ‘igraph’ package [52] to construct social network measures and calculate the minimum reliable window in days that a social metric can be calculated (in our case 365 days were determined). We therefore calculated yearly scores for the social network measure of strength (weighted degree, [53]) at the dyadic level, controlling for the total number of scans of the two dyad members: strength = *N*_ab_/(*N*_a +_ *N*_b_) or the number of times both individuals were within 5 m of each other divided by the total number of scans for which each was the focal animal. Therefore, the Strength metric allows for examination of how frequently a dyad is in association.

We selected this measure as it reflects the strength of the association between the partners, which is the frequency (mean number of associations per unit time) with which the association takes place. These scores were calculated for all dyads of adult females in each group on an annual basis. Hereafter, the dyadic strength score is referred to as the association score.

### (d) Male dominance hierarchy

We constructed male dominance hierarchies to determine which adult male was alpha in multi-male groups. Dominance hierarchies were calculated via Elo rating (See electronic supplementary material for full details [54,55]).

### (e) Statistical analysis

First, to understand if the association patterns we observed were different from that expected by chance, we ran 1000 randomized permutations swapping the partners at random but maintaining the number of interactions for each individual (separately for each group per year). From this, we took an average score (expected association score per dyad per year: Association_exp_) and determined the 95% percentile intervals for the distribution from the 1000 permutations in line with Surbeck *et al*. [27]. Dyads with observed association score (Association_obs_) greater than the expected strength score were observed associating more often than expected by chance. Those with an Association_obs_ greater than the 95% high confidence interval score were considered to be ‘preferred associates'.

To test our predictions, we used a Bayesian multi-level statistical approach for all analysis below. We used the function ‘brm’ from the r package ‘brms’ [56] implemented in r using ‘r-STAN’ [57] to examine association strength, consistency and partner choice. Models were fitted with Hamilton Markov Chains and run in R v.4.1.0 [58]. We present summary statistics for the posterior distribution, including the mean, s.e. and 95% credible intervals (CIs). We used informative priors (normal (0,1) for all continuous variables), four chains and 4000 iterations [56,59]. All *ř* values were < 1.01, which indicates that our models converged, while whole posterior predictive checking examined the goodness-of-fit of the models via the ‘pp_check’ function from the ‘bayesplot’ package [60]. We used the ‘bayes_R2’ function to generate marginal and conditional *R*^2^ values [61].

#### (i) Female associations when the alpha male is present/absent

To examine the similarity of the dyadic association scores in the presence and absence of the alpha male, we used the cosine similarity metric to examine network similarity in primates [62,63]. The cosine similarity metric measures the similarity of associations between two networks and can consider the weight and presence of the associations [64]. It measures the orientation, not the magnitude, such as Euclidean distance of two vectors (*a*,*b*), making it suitable for different sample sizes and is expressed as the cosine of the angle between the two vectors.

$$\cos\theta =\frac{a\cdot b}{\left(||a||b||\right)}.$$

Scores range between 1 (two networks are entirely similar) and 0 (two networks do not share any associations). The cosine values were calculated using the ‘lsa’ package in R [65].

#### (ii) Differences in association patterns

To determine if there were group differences in association patterns in the female gorilla dyads, we examined the association scores. Due to having only one western gorilla group we were unable to directly compare between species and thus run the analysis at the group level. We constructed a general linear mixed model (GLMM: Model 1) with dyadic association score as the response variable and Group as the predictor variable, and total adult group size was included as a control variable.

$$\begin{array}{rl} \mathrm{Model}\;1 & =\;\mathrm{association}\;\mathrm{score}\;∼\;\mathrm{Group}+(1|\mathrm{Dyad})+(1|ID1)\\ & \;+(1|ID2)+(1|\mathrm{Year}) \end{array}$$

#### (iii) Consistency in association patterns

We analysed association consistency in two ways. First, we examined if the associations between dyads were consistent over time. To do so we constructed a GLMM (with beta distribution) that looked at the absolute association score of each dyad as a response variable and included the dyad's association score of the previous year as a predictor variable (Model 2). Initially, we included the interaction between group and association score the previous year but as this did not show a meaningful result, this was removed from the model to fully interpret the fixed effects.

$$\begin{array}{rl} \mathrm{Model}\;2= & \;\mathrm{Association}\;\mathrm{score}\;∼\;\mathrm{Previous}\;\mathrm{association}\;\mathrm{score}\\ & \;+\;\mathrm{Group}+\mathrm{Group}\;\mathrm{Size}+(1|\mathrm{Dyad})\\ & \;+(1|ID1)+(1|ID2)+(1|\mathrm{Year}) \end{array}$$

Second, we determined who the top associate was for each individual each year (Model 3). We then constructed a GLMM with whether each dyad was the top associate for each female that year (Y/N). We included whether the same individual was the top partner the previous year as the predictor variable, and group was included as an additional predictor variable. As above, we removed the interaction and re-ran the model to fully interpret the fixed effects. This analysis was repeated comparing a dyad's association score to their score 2 and 3 years previously.

$$\begin{array}{rl} \mathrm{Model}\;3= & \;\mathrm{Top}\;\mathrm{associate}\;∼\;\mathrm{Previous}\;\mathrm{top}\;\mathrm{associate}+\mathrm{Group}\\ & \;+\;\mathrm{Group}\;\mathrm{Size}+(1|\mathrm{Dyad})+(1|ID1)+(1|ID2)\\ & \;+(1|Year)+(1|\mathrm{DyadNumber}) \end{array}$$

Finally, we examined if preferred associates showed similar consistency in their association duration. To do so we selected a subset of only dyads with a preferred association (*N* = 57). For these dyads, we ran a linear mixed model (LMM: Model 4) with dyadic association score as the response variable and dyadic association score the previous year as the predictor variable. Due to the small number of samples and the majority coming from group KYA, we did not include a group variable in this analysis. This analysis was repeated comparing a dyad's association score to their score 2 and 3 years previously.

$$\begin{array}{rl} \mathrm{Model}\;4= & \mathrm{Association}\;\mathrm{score}\;∼\;\mathrm{Previous}\;\mathrm{association}\;\mathrm{score}\\ & \;+\;\mathrm{Group}\;\mathrm{Size}+(1|\mathrm{Dyad})+(1|ID1)+(1|ID2)\\ & \;+(1|\mathrm{Year}) \end{array}$$

For all models, to control for repeated sampling of the same individuals, dyads and years we included Dyad member *ID*1 and *ID*2 (hereafter ‘*ID*1’ and ‘*ID*2’), Dyad identity (hereafter ‘Dyad’) and year as random effects. For model 3, as we had a score for each dyad member in the dataset per year, this gave two data points per dyad; therefore, we included an additional random effect where each dyad pair per year was given an individual ID number (DyadNumber).

## 3. Results

### (a) Association scores

In total, we collected 95 574 instantaneous scans on three adult female mountain gorilla groups of which 32 958 (34.48%) did not have another adult female within 5 m (table 1 for group comparisons). For one group of western gorillas, we collected 10 435 instantaneous scans of which 7362 (70.55%) did not have another adult individual within 5 m.

**Table 1.** Showing the species and group differences in association scores for mountain and western gorillas. Provided are the mean ± s.d., the range of association scores and the total number of scans, the number of scans when no other female was within 5 m, the number of dyads where the observed score was higher than the mean expected score from 1000 randomized permutations, the number of dyads that were preferred associates and the total number of dyads.

	association score						
gorilla species or group	mean ± s.d.	range	total number of scans	scans with no other female present	number of dyads with score greater than chance	number of preferred associates	total number of dyads
western	0.028 ± 0.018	0.002–0.101	10 435	7 362 (70.55%)	38 (50.67%)	6 (8%)	75
mountain	0.050 ± 0.025	0–0.173	95 574	32 958 (34.45%)	149 (38.60%)	82 (21.24%)	386
breakdown of mountain gorilla groups:							
KYA	0.053 ± 0.030	0–0.173	73 739	22 251 (30.18%)	130 (41.94%)	72 (23.23%)	310
ORU	0.034 ± 0.018	0.002–0.087	12 223	7 382 (60.39%)	19 (41.30%)	10 (21.74%)	46
BIT	0.050 ± 0.013	0.027–0.071	9 612	3 325 (34.59%)	0 (0%)	0 (0%)	30

**Figure 1.**
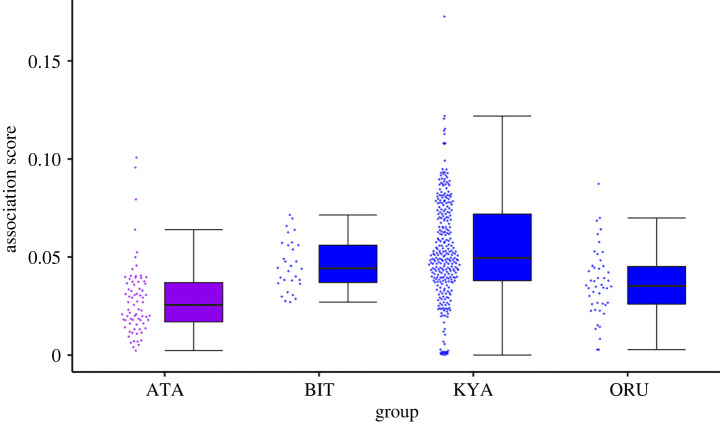
The relationship between female dyadic association scores (*y*-axis) and gorilla group (*x*-axis: western gorilla = ATA (*N* = 75; purple) and mountain gorillas = BIT (*N* = 30), KYA (*N* = 310) and ORU (*N* = 46); blue). The dots represent the distribution of the raw data. See table 2 for the full results of the output of model 1 (*N* = 461 dyads). (Online version in colour.)

We found that there was differentiation in the female–female gorilla association scores as the distribution of association scores were highly variable across groups and years (table 1 and figure 1). Overall, our association scores ranged from 0 to 0.173 with a mean (±s.d.) of 0.047 (±0.025), and there were 187 (of 461: 40.56%) dyads with observed association scores greater than the mean expected association score (see table 1 for a species and group breakdown). Overall, 88 (19.09%) were considered preferred associates (values greater than the upper 95% interval of association values from the randomly generated networks: 8% from western (*n* = 75) and 21.24% from mountain gorilla dyads (*n* = 386); see table 1 for full details). All preferred associates from the western gorilla population were from different dyad pairs but the same individual was a member of four of these dyads. For the mountain gorillas, BIT group had no preferred associates while ORU had 10 from nine different dyads. Group KYA had 72 preferred associates of which the same three individuals were present in 18, 18 and 19 of the dyadic pairings.

**Table 2.** Showing the results of LMM model 1 (*N* = 461 dyads). The estimate, s.e. and 95% CI are provided *R*^2^_CONDITIONAL_ = 0.622 (± 0.022 s.e.) and *R*^2^_MARGINAL_ = 0.040 (± 0.002 s.e.). For all group comparisons, ATA is the reference group. Factors highlighted in italics are considered to have a meaningful effect on the predictor variable (i.e. 95% CI does not intersect with 0).

factor	estimate	s.e.	95% lower CI	95% upper CI
*intercept*	*0.044*	*0.010*	*0.024*	*0.065*
group size	0.001	0.001	−0.002	0.002
*group: BIT*	*0.017*	*0.006*	*0.005*	*0.030*
group: KYA	0.006	0.005	−0.003	0.016
group: ORU	0.009	0.007	−0.004	0.022

#### (i) Female associations when the alpha male is present/absent

To understand if proximity to the alpha male was driving female social relationships, we compared female association scores in the presence and absence of the alpha male by examining the cosine similarity for each group and year separately. We found that for both mountain and western gorillas the scores for cosine similarity for across years were high, indicating that the social networks in the presence and absence of the alpha male were similar (western: 0.969 ± 0.030 (mean ± s.d.), range = 0.927–0.999; mountain: 0.892 ± 0.068, range = 0.713–0.971; KYA: 0.902 ± 0.066, range = 0.713–0.971; BIT: 0.853 ± 0.084, range = 0.762–0.955; ORU: 0.900 ± 0.051, range = 0.832–0.965).

Overall, looking at the mean scores for each species, association scores were higher for females when the alpha male was present for mountain gorillas (present: 0.036 ± 0.021 (mean ± s.d.); absent: 0.020 ± 0.011) and higher in the absence of the alpha male for western gorillas (present: 0.011 ± 0.004; absent: 0.024 ± 0.015). Thus, although association scores were higher for mountain gorillas on average when the alpha male was present (and lower for western gorillas), the scores at the dyadic level were highly similar and suggest that the alpha male is not driving female association scores in our population.

#### (ii) Differences in association patterns

To examine if there was a group-level difference between the association scores, we conducted a GLMM (Model 1, *N* = 461 total dyads and 72 unique dyads) comparing the association score per dyad per year to group. Although the mean value for the mountain gorilla groups was greater than the mean for the western gorilla group (0.050 versus 0.028), not all mountain gorilla groups were different from the western gorilla group. There was a meaningful difference between the mountain gorilla group BIT and the western gorilla group, with BIT showing higher values than the western gorillas and no very low association scores (table 2 and figure 1).

#### (iii) Consistency in association patterns

**Figure 2.**
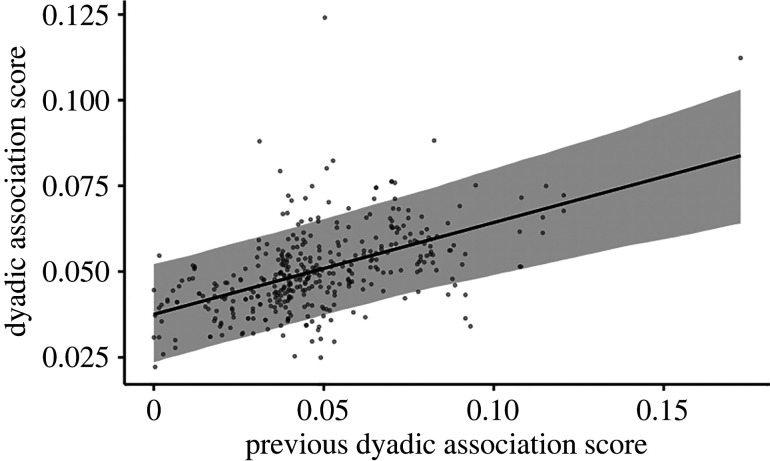
Showing the relationship for female association score between a dyad's current association score (*y*-axis) and their previous association score the year before (*x*-axis). Results are from the output of model 2 (*N* = 385 dyads); for full model results, see table 3. Darker dots are due to data points overlapping.

First, looking at the overall consistency of all dyadic relationships (Model 2, *N* = 385 total dyads and 72 unique dyads), we found that relationships were consistent over time. Those individuals with high association scores in one year were high in the previous year and those with low association scores were low in the previous year (table 3 and figure 2). Additionally, we examined the same relationship from year 1 to year 3 and year 4 but found that the association scores in year 3 or 4 were not predicted by the association scores in year 1 (for results see electronic supplementary material, tables S2 and S3).

**Table 3.** Showing the results of LMM model 2 (*N* = 385 dyads). The estimate, s.e. and 95% credible intervals (CI) are provided. *R*^2^_CONDITIONAL_ = 0.768 (± 0.016 s.e.) and *R*^2^_MARGINAL_ = 0.115 (± 0.030 s.e.). For all group comparisons, ATA is the reference group. Factors highlighted in italics are considered to have a meaningful effect on the predictor variable (I.e. 95% CI does not intersect with 0).

factor	estimate	s.e.	95% lower CI	95% upper CI
*intercept*	*0.037*	*0.007*	*0.023*	*0.052*
*strength score previous year*	*0.267*	*0.049*	*0.173*	*0.364*
*group size*	*−0.004*	*0.001*	*−0.006*	*−0.001*
group: BIT	0.012	0.006	−0.001	0.024
group: KYA	0.004	0.005	−0.007	0.014
*group: ORU*	*0.015*	*0.006*	*0.003*	*0.028*

**Figure 3.**
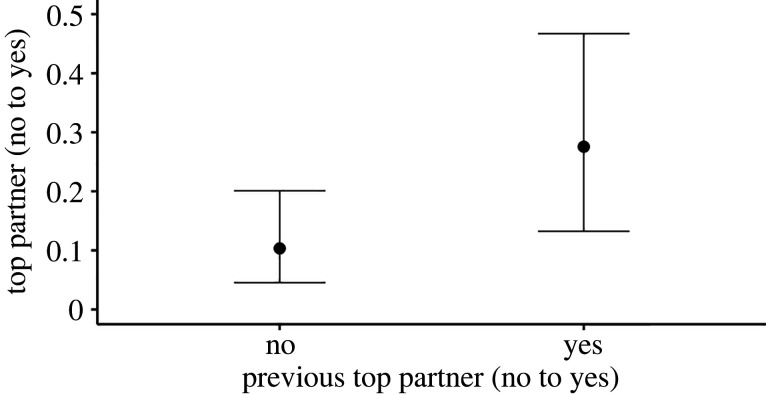
Showing the relationship for whether the dyad partner is the individual's top partner (*y*-axis: predicted probability of being the top partner) and whether the dyad partner was the individual's top partner one-year earlier (*x*-axis: no and yes). Results are from the output of model 3 (*N* = 798 dyads); for full model results see table 4.

Second, we also found that an individual's top partner was consistent over time (Model 3, *N* = 798 total dyads and 72 unique dyads). If an individual's dyad partner was their top association partner in one year, it was most likely they were also their top association partner in the previous year (table 4 and figure 3). Additionally, we examined only those dyads considered to be preferred associates to investigate if these were also consistent for similar or longer periods (Model 4, *N* = 57 total dyads and 26 unique dyads). We found these results echoed those of the top partner analysis, with the scores being consistent across two years but not for 3–4 years (see electronic supplementary material, tables S5–S7 for the statistical analysis).

**Table 4.** Showing the results of GLMM model 3 (*N* = 798 dyads). The estimate, s.e. and 95% CIs are provided. *R*^2^_CONDITIONAL_ = 0.253 (± 0.056 s.e.) and *R*^2^_MARGINAL_ = 0.053 (± 0.023 s.e.). For all group comparisons, ATA is the reference group. Factors highlighted in italics are considered to have a meaningful effect on the predictor variable (i.e. 95% CI does not intersect with 0).

factor	estimate	s.e.	95% lower CI	95% upper CI
*intercept*	*−2.172*	*0.422*	*−3.044*	*−1.380*
*top partner previous year: yes*	*1.194*	*0.268*	*0.679*	*1.721*
group size	−0.318	0.496	−0.866	0.223
group: BIT	0.405	0.574	−0.719	1.533
group: KYA	0.028	0.452	−0.840	0.937
group: ORU	0.249	0.496	−0.718	1.221

## 4. Discussion

This study compares long-term association patterns in mountain and western gorillas for the first time, and it is one of very few to compare association patterns directly between species. Overall, we found that females in the western gorilla group were more likely to be further than 5 m from any other adult female (70.55% of scans) compared to females in the mountain gorilla groups (34.48% of scans). When examining the overall association scores between species, western gorillas showed lower (52% lower) mean association scores than mountain gorillas. However, we found no statistical difference between the species, which may be due to intra-specific variation among the three groups of mountain gorillas. Further examination of additional western gorilla groups in the future will help to differentiate group- and species-level differences; however, this requires substantial financial and temporal resources due to the difficulty in habituating western gorillas. We found a range of dyadic association scores in both species, indicating differentiation in association patterns, with over a third of mountain and half of western gorilla dyads associating more often than expected by chance. Although a greater proportion of western gorilla dyads associated more often than by chance, there was a twofold increase in the number of preferred associates in mountain gorilla groups compared to the western gorilla dyads when controlling for total dyad number. For both species we found associations and top partners to be consistent across a maximum of 2 years, but not for longer time periods (although it is worth noting that small sample size in the analysis at 3–4 years may have affected the power of the analysis).

Social relationships between the alpha male and adult females are the foundation of gorilla groups [40]. Because male–female and female–female relationships are not isolated from each other (e.g. [66]), we examined the role of the alpha male in female association patterns. We found that female dyadic associations are not a consequence of the females' proximity to the alpha male because dyadic association scores in his presence and absence were similar. Interestingly, the scores in the presence of the alpha male were generally lower for western than mountain gorillas and similar for both species in his absence. The observed differences between the mountain gorilla groups and the western gorilla group might be related to the differences in food availability and distribution between the species and the western gorillas’ maintaining greater distance from others as a means to reduce feeding competition [67]. Mountain gorillas tend to come together and rest in close spatial proximity and then spread out more when they feed [40,44], whereas western gorillas are even more spatially dispersed when they feed (M. Robbins 2022, pers. obs). For mountain gorillas, the abundant and uniform food distribution may lead to the alpha male and females being in proximity to each other more than western gorillas, regardless of whether they are feeding or resting. As western gorillas' food resources are more dispersed, this may lead to each female spending on average less time in spatial proximity to the alpha male and each other (70.55% of western gorilla scans did not have another adult female within 5 m compared to 34.48% for mountain gorillas). However, higher mean association scores between females in the alpha's absence may indicate that they prefer certain individuals in his absence but are less selective in their association partner in his presence. Further analyses of patterns of male–female associations in gorillas and other species are needed to understand the role of males in female–female associations and the relative importance of reducing feeding competition versus mechanisms of predator avoidance.

Gorilla associations in our populations were consistent for relatively short time periods (2 years on average) in comparison to other species, such as chimpanzees, where strong female relationships can exceed 4 years [20,21]. Gorilla females disperse between social groups multiple times in their lives, which differs from most species with female-biased dispersal. The strength of social relationships in other female dispersing species tends to be long-term and stable [20,23,25–27] but can be very weak [19]. In the species that exhibit strong associations, females may build similar relationships to those of philopatric females due to the potential for long-term relationship stability. Therefore, females may invest in a few strong relationships and obtain the benefits of improved cooperation, health and fitness, although the underlying mechanisms driving this relationship formation will likely differ from that of philopatric females (i.e. not kin-related benefits [3]). However, as gorillas transfer between groups multiple times in their lives, such long-term stability in group membership is a rare commodity because groups disband if the alpha male dies and females without dependant infants emigrate to another group. As a result, females may not have the opportunity to invest heavily in long-term associations. Alternatively, even if they reside in the same group for many years, due to the possibility that their partner could disperse, the risk of investing heavily in one partner only to be abandoned may be too great to allow for long-term, consistent strong associations to form. Examining if the duration of co-residency influences female associations will help elucidate why we see the variability in our study groups. Overall, the rare gorilla social system with secondary dispersal may have led gorillas to be more flexible in their partner choices and association patterns.

Strong associations between females may not be a primary factor driving their reproductive success. Voluntary dispersal to smaller groups to avoid feeding competition [15] or investment in strong relationships with the alpha male rather than with other females (for protection from other males and predators), with a preference for larger males [68,69], is more likely to have a positive impact on fitness. Additionally, if the sole silverback dies (one-male groups being the norm in western gorillas and approximately 50% of mountain gorilla groups), females are forced to find another silverback (involuntary dispersal). In western gorillas, infant mortality is higher as the alpha male ages, negatively impacting female reproductive success and linked to increased female dispersal as males age [15,70]. Therefore, female gorillas may focus more on their relationships with males and remain flexible in their female associations due to the dynamic, ephemeral nature of their social groups. This is reflected in our observations of fewer preferred associates in western gorillas (which have higher rates of group disintegrations and female dispersal) and the most preferred associates in the mountain gorilla group with the longest observation time (KYA: over 15 years). Similarly, grooming, a common metric of affiliative relationships, among adult females has almost never been observed in western gorillas [45] and cooperation has not been observed. Nonetheless, female gorillas do form preferred associations, suggesting they provide some benefits. To better understand the dynamic nature of female association patterns, future studies should investigate other behaviour patterns including agonistic relationships as well as demographic factors (e.g. alpha male tenure and female co-residency duration). Alternatively, it may be that female association patterns are driven by certain short-term characteristics such as preferences for food resources or if females have a dependent infant, and such homophily could lead to greater short-term association that we observe.

In conclusion, we find that female mountain and western gorillas form dyadic association patterns that were consistent for a few years and variable in strength. Due to the rare dispersal pattern in the gorilla genus, it may be that flexibility is key for association between females. Such flexibility and uncertainty in social change may be as cognitively challenging as tracking social interactions between a few preferred associates [11,71]. Forming long-term, consistent, stable associations might not be the best strategy for females due to the constant possibility of voluntary or involuntary dispersal of social partners. We found that the alpha male is not the main driver of these female–female associations in both species. Females who invest in preferred associations may reap benefits of enhanced infant survival via reduced predation risk or increased defence of resources. This study therefore highlights a need to understand the exact benefits female gorillas gain from these preferred associations and what other factors constrain and promote associations and shape preferences. Examining female–female social relationships in a species with bi-sexual dispersal is rare in mammalian sociality and so our research adds to the general knowledge of how social relationships are formed and maintained in a flexible society. Understanding behavioural patterns of extant great apes, such as the interplay between dispersal patterns and social interactions, as well as the variability exhibited, is useful to consider when examining patterns among the same variables in modern humans.

## Data Availability

Data and code are available are provided in the electronic supplementary material [72].
